# A Community‐Engaged Approach to Identifying and Addressing Viral Hepatitis Determinants in Michigan Asian American Communities

**DOI:** 10.1111/jvh.70149

**Published:** 2026-02-06

**Authors:** Parnnate Wongsirisakul, Neehar D. Parikh, Jonathan Troost, Hannah Par, Thanvir Chowdhury, Qingqing Zhang, Yi‐Chun Wang, Tsu‐Yin Wu, Ponni V. Perumalswami

**Affiliations:** ^1^ Division of Gastroenterology and Hepatology University of Michigan Ann Arbor Michigan USA; ^2^ Michigan Institute for Clinical and Health Research USA; ^3^ School of Public Health University of Michigan Ann Arbor Michigan USA; ^4^ Center for Health Disparities Innovations and Studies Eastern Michigan University Ypsilanti Michigan USA

**Keywords:** Asian American, community engagement, hepatitis B virus, hepatitis C virus, liver cancer

## Abstract

Approximately 75% of people infected with the hepatitis B virus (HBV) and hepatitis C virus (HCV) in the United States (U.S.) have yet to be tested, thus leading to the risk of liver disease progression that can be prevented by early diagnosis. Asian Americans (AA) are disproportionately infected with HBV and HCV in the U.S., including in the state of Michigan. Using a theory‐informed approach, we conducted a bi‐level quantitative study to identify determinants of viral hepatitis and liver cancer care and treatment. We drafted and administered surveys to 151 community members across three Michigan AA communities (Burmese, Chinese and Bangladeshi). The results were then presented to a Community Advisory Panel (CAP) comprised of community leaders who suggested interventional adaptations. Survey respondents who had previously tested for viral hepatitis were wealthier, more proficient in English and had immigrated to the U.S. earlier. They also had higher health literacy, more knowledge of HBV transmission and greater self‐efficacy. CAP members recommended that education be delivered in a shareable video format to address health literacy and medical access. Additional recommendations included tabling at community events and tailoring programmes to age. Using these data, we can develop a needs‐based, culturally targeted intervention to raise awareness, reduce stigma and increase viral hepatitis screening in Michigan AA communities.

**Abbreviations**
AA: Asian American
HBV: hepatitis B virus
HCV: hepatitis C virus

## 1 Introduction

Currently, Asian Americans (AAs) make up 7% of the United States (U.S.) population, but they account for more than half of the viral hepatitis B (HBV) cases [1]. Similarly, viral hepatitis C virus (HCV) is endemic in many Asian countries [2]. Viral hepatitis infections are usually asymptomatic and can result from unprotected sexual intercourse or bloodborne interaction with a carrier [3, 4]. Regardless of screening recommendations from the Centers for Disease Control and Prevention (CDC), approximately 75% of all persons living with HBV and HCV in the U.S. have not yet been tested [5, 6]. There are several treatments available to cure HCV or control HBV, which decreases the risk of liver disease progression and hepatocellular carcinoma development. However, gaps in diagnostic testing in patients with HCV or HBV mitigate these benefits.

To explore the determinants of viral hepatitis infection and liver disease progression in immigrant Asian populations, we launched REducing AAPI Community Health disparities for LIVER Cancer in MIchigan (REACH‐LIVER MI). REACH‐LIVER MI is structured as a bi‐level, mixed‐methods study in three Michigan AA communities. These communities were identified as having high hepatitis rates and low engagement in health‐seeking behaviours [7]. We have previously developed, implemented and evaluated viral hepatitis interventions in West African communities in New York City with the Hepatitis Outreach Network (HONE). HONE has led to HBV and HCV screening (*n* = 7065, 6%–9% viral hepatitis prevalence) with high linkage to care (> 85%) [8]. Adaptation of the HONE model can reach additional underserved communities, including those that are culturally and geographically different.

We have previously reported themes that emerged from the qualitative portion of the study [9]. The focus group and interview transcripts from community leaders and external stakeholders revealed common and distinct barriers and facilitators of viral hepatitis screening and treatment between communities. Herein, we aimed to supplement these qualitative findings with quantitative results from our survey of community members, with the aim of creating a holistic view of three Michigan AA communities and their perspectives on viral hepatitis to understand how we can best adapt a culturally tailored intervention.

## 2 Materials and Methods

In this study, we developed a multi‐level community‐based survey for AAs in Michigan, including communities with poorly characterised healthcare beliefs (Burmese and Bengali), to identify key determinants of viral hepatitis and liver cancer testing and treatment. The first level engages external stakeholders from public health, cancer coalitions and Asian diaspora associations in a focus group, and the second level involves community leader interviews and community member surveys. A more detailed description can be found elsewhere [9]. We reviewed the survey results with community leaders and used the evidence‐based implementation science Framework for Reporting Adaptations and Modifications‐Expanded (FRAME) [10] to adapt and tailor the HONE process to Michigan AA communities.

### 2.1 Conceptual Model

Ajzen's Theory of Planned Behaviour (TPB) was developed to explain how beliefs link to behaviour. Since its conception, the TPB has been widely applied to the public health sector, especially in understanding cancer screening behaviours. Our prior work has adapted TPB to viral hepatitis and liver cancer testing and treatment [8, 11]. In the TPB, intent to complete an action or behaviour is characterised by three factors: attitude towards the behaviour, subjective norm and perceived behavioural control [12]. More specifically, we are considering one's attitude towards HBV and HCV screening, hepatitis knowledge, beliefs and stigma and perception of ease/difficulty in accessing screening. Health attitudes are largely based on education, social support and past behaviours.

### 2.2 Data Collection

Leaders from our three target AA communities—the Burmese community in West Michigan, the Bangladeshi community in Hamtramck, MI and the Chinese community in Detroit, MI—facilitated the recruitment of survey participants by phone, small in‐person gatherings, email and word of mouth. Eligible individuals were required to: (1) be at least 18 years old; (2) self‐identify as AA and (3) speak English, Burmese, Bangladeshi or Chinese. Once identified, trained bilingual research staff administered the surveys. Written and informed consent of participants was obtained in person at community sites, by phone or using a virtual format as advised by community leaders.

The surveys were translated using a certified translation service, with back translation by the bilingual field coordinators. Each survey was completed within an hour, and participants received an incentive. Fifty Burmese, 50 Bangladeshi and 51 Chinese community members were surveyed. To assess diversity of responses, recruitment was targeted so that participants were half men/women and half below/above 50 years of age for each community. Participants were given the choice to complete the survey in English or one of the target population languages. The data was stored in REDCap. All research activities were reviewed and approved by the University of Michigan Institutional Review Board prior to study initiation.

### 2.3 Survey Development

The survey was developed from a variety of validated sources, along with the points raised by community leaders and external stakeholders. Of the barriers expressed by community leaders, health literacy, racism and medical mistrust were prevalent, so such topics were included in the survey. These questions were derived from articles specifically involving racism [13] and health literacy [14] in AAs, as well as medical mistrust associated with cancer screening [15]. Several survey questions regarding demographics, language preference/English proficiency, insurance, healthcare utilisation and food insecurity were adapted from the California Health Information Survey [16]. Additionally, we asked community members about their current cancer screening status and health‐seeking behaviours [17, 18, 19]. The bulk of the survey assessed knowledge, attitudes and beliefs on viral hepatitis and liver cancer as outlined in a previous publication that used the Health Behaviours Framework to measure HBV testing [20].

### 2.4 Analysis

For variables such as perceived susceptibility and severity, barriers to HBV testing, self‐efficacy, health literacy, racism and group‐based medical mistrust, community members chose a statement that corresponded to a number. The self‐efficacy and medical mistrust sections were scored on a 5‐point Likert scale (5 = strongly agree, 1 = strongly disagree). Specifically, the group‐based medical mistrust score was divided into three sections: suspicion (6–30), group disparities in healthcare (3–15) and lack of support from healthcare providers (3–15) [21]. The average scores of viral hepatitis screening determinants can be found in Table 3. The HBV knowledge of transmission score was the sum of the number of questions correctly answered, from 0 to 11. Perceived susceptibility and severity were both on a scale from 0 to 6. Self‐efficacy ranged from 12 to 60 and racism ranged from 5 to 30. Only three categories, barriers to HBV testing (8–24), health literacy (2–8) and group disparities in healthcare (3–15), had an inverted score, meaning that lower scores equaled more concern about barriers, higher health literacy and more healthcare disparities respectively.

### 2.5 Community Advisory Panel

**FIGURE 1:**
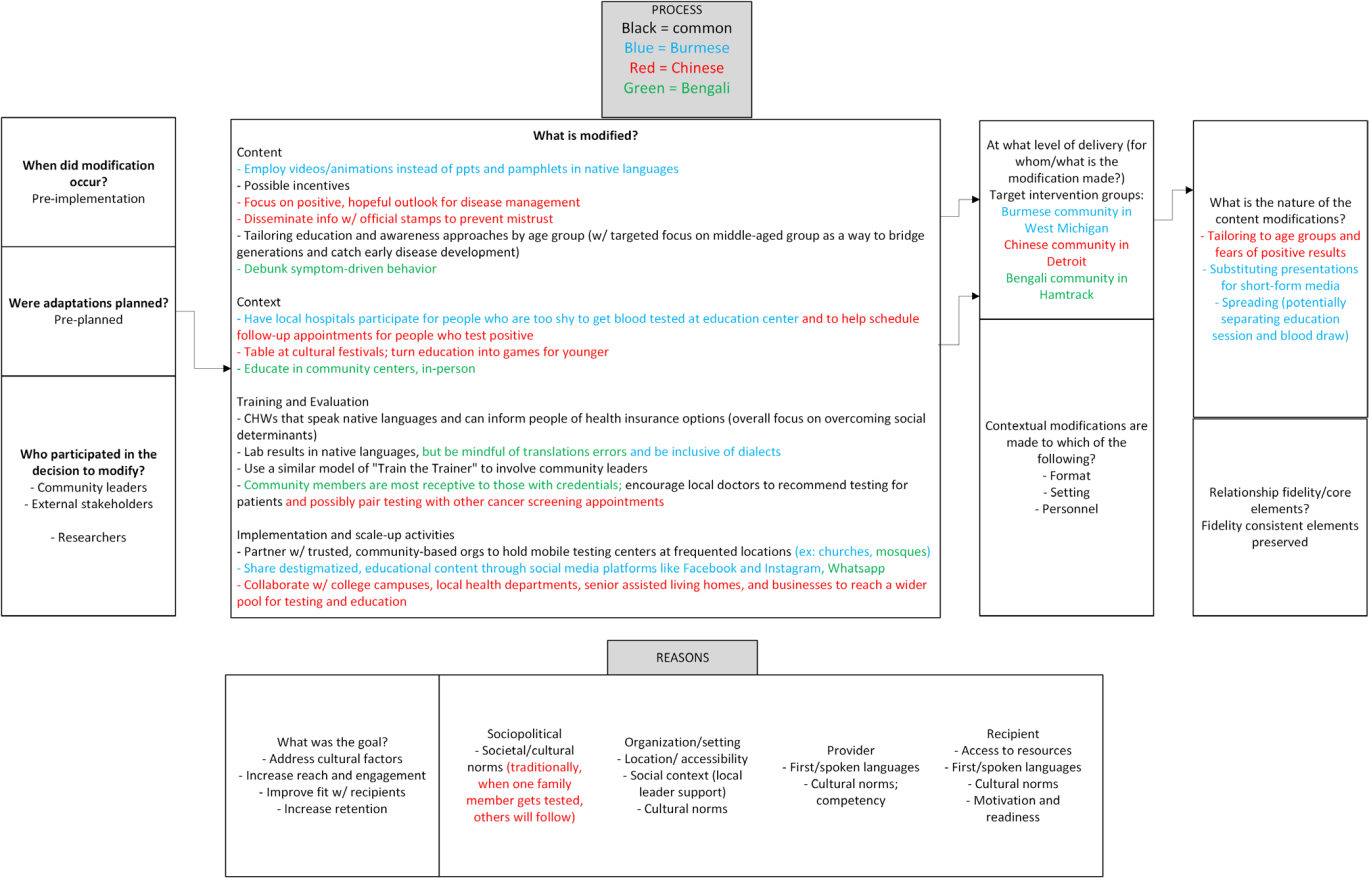
FRAME diagram based on input from Michigan Asian American Community Leaders. The process and reasons behind modifying a viral hepatitis screening and educational intervention are depicted in the figure. Each suggestion is colour‐coded, with black representing common ideas across the Burmese, Chinese and Bengali communities. Blue, red and green represent the Burmese, Chinese and Bengali‐specific factors respectively.

Community Advisory Panels (CAPs), an evidence‐based implementation strategy [22], were established in the first phase of the study [9] to successfully develop and execute this project. Community leaders (*n* = 3–5 leaders) were invited from each of the three AA communities. The CAPs had shared leadership and compensated effort. Two CAP focus groups were held—one to review the survey findings and the other to collaborate on factor prioritisation, assigning impact scores and mapping implementation strategies. The modifications of the HONE model and the necessities of each community were colour‐coded and documented using FRAME (see Figure 1). The FRAME is ideal for this study as it considers what is modified, the nature of the modification (content, evaluation and training) and the rationale for the modification [10].

## 3 Results

### 3.1 Sociodemographic Characteristics

The sociodemographic data comparing the communities can be found in Table 1. Out of the three communities, the Chinese community had the oldest members and the members who had immigrated to the U.S. earliest. In contrast, the Burmese community was the youngest, with a median age of 15.5 years younger than the Chinese community. However, the Bengali community was the latest to immigrate, as many members reported a U.S. immigration date 9 years later than the Chinese community. The greatest percentage of Burmese community members had a household income between $75,000 and $99,999 (48%), received at least a high school graduation (47%), were insured through their employers (82%), and spoke English well (53%). Most of the Chinese community was also employer‐insured (59%) and proficient in English (74%), but more Chinese respondents were college‐educated (73%) and had a higher household income of over $100,000 (45%). In comparison, many Bengali respondents had a household income of less than $35,000 (42%), received less than a high school graduation education (60%), were insured through Medicaid (64%) and were not proficient in speaking English (54%).

**TABLE 1:** Sociodemographic characteristics, familiarity with viral hepatitis and cancer screening engagement of Michigan Burmese, Bengali and Chinese communities.

	Burmese (*n* = 50), *n* (%)	Bengali (*n* = 50), *n* (%)	Chinese (*n* = 51), *n* (%)	*p*
Median age [IQR]	34.5 [25, 45]	48 [39, 58]	50 [39, 67]	< 0.001
Sex, female	28 (56)	25 (50)	27 (53)	0.83
Employed	34 (68)	30 (60)	36 (71)	0.50
Household income				< 0.001
< $35,000	0	21 (42)	10 (20)	
$35,000–$49,999	2 (4)	18 (36)	6 (12)	
$50,000–$74,999	10 (20)	6 (12)	5 (10)	
$75,000–$99,999	24 (48)	0	5 (10)	
> $100,000	14 (28)	0	23 (45)	
Education				< 0.001
Less than high school graduation	3 (6)	30 (60)	6 (12)	
High school graduation	23 (47)	11 (22)	8 (16)	
Some college	23 (47)	9 (18)	37 (73)	
Insurance				< 0.001
Medicaid	6 (12)	32 (64)	1 (2)	
Medicare	6 (12)	1 (2)	17 (33)	
Employer‐provided	41 (82)	1 (2)	30 (59)	
Out‐of‐pocket	0	9 (18)	1 (2)	
Uninsured	1 (2)	8 (16)	1 (2)	
Would you say you speak English…				< 0.001
Very well/well	26 (53)	15 (30)	37 (74)	
Not well	13 (27)	27 (54)	9 (18)	
Not at all	10 (20)	8 (16)	4 (8)	
Median date of U.S. immigration [IQR]	2011.5 [2008, 2013]	2017 [2016, 2019]	2008 [1991, 2013]	< 0.001
Before today, I had heard about HBV/HCV screening	13 (26)	23 (46)	30 (59)	0.004
Yes, I have been tested for HBV	10 (20)	5 (10)	15 (29)	0.05
If I have not, I am likely to get screened for HBV in the next 2 months	1 (3)	3 (8)	5 (20)	0.04
Yes, I have been tested for HCV	8 (16)	1 (2)	7 (14)	0.05
If I have not, I am likely to get screened for HCV in the next 2 months	1 (3)	4 (10)	4 (13)	0.35
How many of your friends and family members get tested for HBV?				0.09
Most of them	5 (10)	5 (10)	11 (22)	
Some of them	9 (18)	9 (18)	18 (35)	
None of them	36 (72)	36 (72)	22 (43)	
Cancer screening history, not tested^a^				
Breast	5 (45)	10 (48)	7 (32)	0.63
Cervical	5 (18)	15 (60)	3 (11)	< 0.001
Colorectal (PSA test)	13 (65)	12 (92)	10 (77)	0.58
Colorectal (stool test)	30 (81)	21 (100)	19 (90)	0.19
Prostate	36 (97)	20 (95)	17 (81)	0.07

^a^ Adjusted for recommended testing age and sex.

Of the 151 participants, 32 received previous testing for viral hepatitis and 119 had not. To understand what differences may exist, we analysed the survey determinant data based on viral hepatitis screening status. When the sociodemographic results are viewed through HBV/HCV testing history (see Table 2), receiving some college, having employer‐provided health insurance and speaking English well were the most common answers. In the never tested group, a higher proportion was in the lowest income bracket (22%), in contrast to the 44% of previously tested participants who reported income of over $100,000. Date of U.S. immigration marked another difference as the never tested group immigrated an average of 3 years later (standard deviation 3.06).

**TABLE 2:** Sociodemographic characteristics, familiarity with viral hepatitis and cancer screening engagement, divided by viral hepatitis testing history.

	Never tested (*n* = 119), *n* (%)	Previously tested (*n* = 32), *n* (%)	*p*
Median age [IQR]	44 [30, 59]	44.5 [37.5, 54.5]	0.46
Sex, female	64 (54)	16 (50)	0.70
Employed	80 (67)	20 (63)	0.62
Household income			0.10
< $35,000	26 (22)	5 (16)	
$35,000–$49,999	20 (17)	6 (19)	
$50,000–$74,999	19 (16)	2 (6)	
$75,000–$99,999	26 (22)	3 (9)	
> $100,000	23 (19)	14 (44)	
Education			0.15
Less than high school graduation	31 (26)	8 (25)	
High school graduation	37 (31)	5 (16)	
Some college	50 (42)	19 (59)	
Insurance			
Medicaid	33 (28)	6 (19)	0.30
Medicare	17 (14)	7 (22)	0.30
Employer‐provided	52 (44)	20 (63)	0.06
Out‐of‐pocket	9 (8)	1 (3)	0.37
Uninsured	10 (8)	0	0.09
Would you say you speak English…			0.22
Very well/well	57 (49)	21 (66)	
Not well	42 (36)	7 (22)	
Not at all	18 (15)	4 (13)	
Median date of U.S. immigration [IQR]	2014 [2008, 2016]	2011 [2005.5, 2014.5]	0.12
Before today, I had heard of HBV/HCV	87 (73)	31 (97)	0.004
Before today, I had heard about HBV/HCV screening	39 (33)	27 (84)	< 0.001
I have been tested for HBV	0	30 (94)	< 0.001
If I haven't, I am likely to get screened for HBV in the next 2 months	8 (9)	1 (100)	0.01
I have been tested for HCV	0	16 (50)	< 0.001
If I haven't, I am likely to get screened for HCV in the next 2 months	8 (8)	1 (10)	0.87
How many of your friends and family members get tested for hep B?			< 0.001
Most of them	5 (4)	16 (50)	
Some of them	25 (21)	11 (34)	
None of them	89 (75)	5 (16)	
Cancer screening history, not tested^a^			
Breast	19 (44)	3 (27)	0.11
Cervical	22 (34)	1 (6)	0.01
Colorectal (PSA test)	25 (71)	10 (91)	0.69
Colorectal (stool test)	56 (89)	14 (88)	0.87
Prostate	60 (95)	13 (81)	0.06

^a^ Adjusted for recommended testing age and sex.

**TABLE 3:** Average scores of HBV/HCV screening determinants.

	Burmese	Bengali	Chinese	*p* from communities	Never tested	Previously tested	*p* from testing history
Knowledge of transmission, possible range 0–11	7.1	6.9	7.9	0.003	7.1	8.1	0.003
Perceived susceptibility, range 0–6	3.2	2	2.6	< 0.001	2.5	3	0.05
Perceived severity, range 0–6	4.3	4.4	3.8	0.01	4.2	4.2	0.77
Barriers to HBV testing, range 8–24^a^	13	14.4	11	< 0.001	13.1	11.5	0.02
Self‐efficacy, range 12–60	46.5	38.8	49.2	< 0.001	43.6	49.7	< 0.001
Health literacy, range 2–8^a^	4.9	4.8	3.4	< 0.001	4.5	3.8	0.11
Racism, range 5–30	5.8	5.2	5.2	0.77	5.5	5.1	0.23
Group‐based medical mistrust							
Suspicion, range 6–30	11.9	8.9	11	< 0.001	10.6	10.6	0.99
Group disparities in healthcare, range 3–15	11.2	12.8	11.8	< 0.001	12	11.9	0.60
Lack of support from healthcare providers, range 3–15	7.6	4.7	7.1	< 0.001	6.3	7	0.20

^a^ Where a lower score respectively indicates more concern about HBV barriers and high health literacy.

### 3.2 Familiarity With Viral Hepatitis

Nearly all Chinese participants had heard of HBV/HCV (98%) and the majority had heard of HBV/HCV screening (59%). In comparison, 76% of Bengalis and 60% of Burmese were familiar with viral hepatitis and ever fewer were familiar with its screenings (46% Bengali and 26% Burmese). 29% of Chinese, 10% of Bengali and 20% of Burmese community members had tested for HBV, with many being between the ages of 40–49. For HCV, 14% of Chinese, 2% of Bengali and 16% of Burmese community members had been tested, with the common testing age being from 30 to 39 years old. For respondents who had no prior history of HBV/HCV screening, Chinese participants self‐reported an increased likelihood of getting screened in the next 2 months, and Burmese participants had the lowest likelihood. For respondents that had tested for viral hepatitis in the past, they generally had higher rates of engagement with other cancer screenings (see Table 2). Another notable feature is that most of the previously tested participants had social support, as their friends and family had also been tested/suggested viral hepatitis testing.

### 3.3 Cancer Screening Engagement

Reports of cancer screening status and behaviours were corrected based on sex and age‐appropriate screening recommendations. Across breast, cervical, colorectal (PSA and stool test) and prostate cancer screenings (see Table 1), the Bengali community appears to have the least number of members who had a previous history of testing behaviour, while the Chinese community had the most. When asked why they had not completed the exams, Bengali community members were more likely to say that they had never given cancer screening any thought beforehand, whereas Burmese and Chinese community members regularly replied that their doctors did not tell them they needed it.

### 3.4 Measured Determinants of HBV/HCV Screening

Notable differences can be observed between the communities across all categories except perceived severity and racism. Bengali respondents appeared to have the least knowledge of HBV transmission routes (6.9) and the lowest perceived susceptibility (2) as opposed to Chinese respondents, who had the most knowledge (7.9) and Burmese respondents, who had the highest perceived susceptibility (3.2). Burmese community members also reported the greatest suspicion (11.9), lack of support from healthcare providers (7.6) and most group disparities in healthcare (11.2). In contrast, Bengali community members had the lowest suspicion (8.9), lack of support (4.7) and felt the least group disparities in healthcare (12.8). Meanwhile, most Chinese community members were not concerned about potential barriers to HBV testing. Overall, they had the highest self‐efficacy (49.2), whereas Bengali participants had the lowest (38.8).

Between those who had previously tested for viral hepatitis and those who had not (see Table 2), there were no significant differences between perceived severity, suspicion and group disparities in healthcare. Respondents with HBV and HCV testing history had a slightly higher perceived susceptibility (3) and experienced slightly less racism (5.1). They also reported greater health literacy (3.8). Significant differences were observed across knowledge, barrier and self‐efficacy scores. Respondents with testing history knew more about HBV transmission (8.1) and reported that they faced more barriers, but they had a stronger belief in their capacity to get screened if desired (49.7).

### 3.5 Community Advisory Panel (CAP) Recommendations

After discussing the survey results, the CAPs suggested modifications to HONE. In the original HONE design, mobile testing and education delivered by bilingual community health workers were paired at community centers. To capitalise on strong family structures within certain communities and reach all age groups, CAP leaders recommended that educational programmes target middle‐aged adults. Ideally, their elderly parents would look to them for advice and their young children would be more willing to receive their instruction. CAP leaders, specifically from the Burmese and Chinese communities, also proposed an option where education and screening are not always paired. Instead, it was recommended that people receive education, then be given the option to screen at a local hospital/clinic later. Burmese leaders argued that some may not want to be tested in a location full of familiar faces due to the stigma of a positive infection, whereas Chinese leaders thought it best if HBV/HCV screenings were paired with cancer screenings in a hospital setting, where people are more likely to follow up.

In addition to offering separate screening and education options, Burmese CAP leaders requested educational videos instead of presentations or pamphlets. Burmese community members reported the lowest health literacy and English proficiency. Furthermore, several different dialects exist for written language within the Michigan Burmese community. Thus, it would be easier to teach people through visual means, especially since videos can be shared through social media platforms. Burmese leaders explained that their community is well‐connected through various apps such as Facebook and YouTube where they already post health content. Using the Internet and social media to access health information tends to be more common in minority populations [23] as it can offset other disadvantages like rurality and language barriers.

For the Chinese community, many of the barriers to HBV testing they experience are intrinsic (stigma, medical mistrust) instead of extrinsic (transport, cost), possibly due to how long the members have lived in the U.S. or the greater number of resources that come with settling in a large city like Detroit. To address high suspicion scores and prevent misinformation, Chinese CAP leaders advised that the educational materials include obvious endorsements from validated health organisations so that people may fact‐check the data. In contrast, Bengali community members reported having received strong support from healthcare professionals and would theoretically respond better to educators with medical credentials. Therefore, Bengali CAP leaders recommended in‐person, presentation‐style education led by experts in viral hepatitis for the most effective education.

## 4 Discussion

On average, the Burmese community was comprised of younger members with lower health literacy. Meanwhile, Bengali community members were the latest to immigrate to the U.S., often had fewer years of education, and were most likely never screened for any cancers or viral hepatitis. In contrast, Chinese community members had been in the U.S. the longest, reported the highest household income, and noted the least number of barriers to HBV/HCV screening. Overall, respondents who had previously tested for viral hepatitis were wealthier, spoke English better and immigrated to the U.S. earlier. They also had higher health literacy, more social support, more knowledge of HBV transmission and greater self‐efficacy.

Among predisposing determinants of viral hepatitis vaccination and screening, sex and age of immigration have important policy implications. Being pregnant while living in the U.S. increases the chances of HBV screening, as testing is part of routine prenatal care. Considering the average ages of which survey respondents immigrated to the U.S.—21 for Burmese, 33 for Chinese and 40 for Bengali community members—it is plausible that some women from each community had children and were screened while in the U.S. Similarly, in the U.S., infants have been routinely vaccinated for HBV since 1991 [24], but only six respondents were born in the U.S. For those born in Myanmar, HBV infant immunisations were adopted in 2003 but paused after 2009 due to funding problems, then reinstated in 2016. In Bangladesh, HBV vaccinations began in 2003 as well and were widely effective [25]. China began birth dose vaccinations in 1985, but the cost made it exclusive until the government stopped charging in 2005 [26]. With the ages of our participants, it is likely that very few were vaccinated for HBV in their birth countries.

In addition to national screening and vaccination policies, the potential role of health insurance warrants attention. The majority of Chinese respondents reported to be covered by employer‐provided insurance along with Medicare. Medicare Part B covers HBV and HCV testing if pregnant, prone to blood exposure/infection or high risk (i.e., current/past history of injection drug use, had received a blood transfusion prior to 1992, born in a country with high prevalence). If not high risk, one HCV screening is covered if born between 1945 and 1965. In the Bengali community, many are on Medicaid or undocumented and uninsured. In Michigan, Medicaid covers HBV testing for those under 21 and HCV testing for all as part of the 2021 We Treat Hep C initiative. Because of policy restrictions, confusing terminology for those who speak English as a second language, and/or lack of awareness of policy benefits, survey participants may not capitalise on their screening eligibility.

This unfamiliarity with insurance coverage, or cost of medical services in general, can also impact the extent to which community members partake in health‐seeking behaviours. Members of the Bengali community reported the lowest participation in cancer screenings, which could be a result of having the latest U.S. immigration date and low English fluency. They may have had less time to adjust to the U.S. healthcare system or may fear interacting with the medical system entirely due to their legal status as undocumented immigrants, have been found to engage in healthcare services far less than documented immigrants [27]. Although the Burmese respondents did not immigrate to the U.S. the latest, they did immigrate the youngest. They may not be comfortable navigating the healthcare system on their own or may lack a social network to ask for medical advice outside of their doctors, which is problematic given that the Burmese community had the highest mistrust in providers.

Of the three communities, the Chinese community seemed most active in cancer and viral hepatitis screenings. This finding is consistent with the hypothesis that people who previously engage in cancer screening are open to receiving other preventative health measures [28]. In addition, more Chinese respondents report that some or most of their friends and family members have previously been tested for HBV and have suggested that they get tested as well. The weight of the words and actions of their inner circle was also echoed in focus groups with Chinese community leaders, as well as studies on HBV screening and Chinese social norms [29]. However, the number of Chinese participants who had heard of viral hepatitis screening in some capacity was much greater than the number who completed screening. The same can be said for the Burmese and Bengali communities. Considering that each community had high self‐efficacy, the discrepancy in screening awareness and execution may be attributed to a lack of viral hepatitis education.

The desire to be screened can be increased by learning more about screening benefits, addressing social determinants of health and reducing stigma associated with infection. According to the survey results, 76% of respondents report that they think that people infected with HBV or HCV are avoided. Furthermore, members from all three communities expressed at least slight concern about bringing shame to their families or burdening their families with their illness. One of the most cited barriers to HBV testing for Burmese and Bengali participants was even the fear of positive infection. Whether that fear was a product of stigma, treatment costs and/or disease trajectory, apprehension can hopefully be lessened by comprehensive educational campaigns and eliminating the anxiety associated with the unknown. Specifically, Chinese CAP leaders supported casting disease outlook in an optimistic light in educational materials so that people can remain hopeful about their health. For Bengali CAP leaders, debunking symptom‐driven behaviour was most salient.

Regarding education, knowledge of transmission routes must also be emphasised, especially since previously tested individuals presented higher knowledge scores. Evident from the distribution of answers, most respondents understand that viral hepatitis can be blood transmitted, but they also incorrectly believe in saliva transmission. 64% of participants think that HBV can be spread by sharing eating utensils, 55% assume that infection travels when someone coughs/sneezes and 60% believe that sharing cigarettes is an HBV risk factor. Because knowledge and self‐efficacy scores varied significantly, we conclude that viral hepatitis awareness and education are a necessary first step to providing care and control. In theory, if an educational programme were to be delivered, the increase in HBV knowledge would also increase community members' perceived susceptibility and severity.

There are several strengths and limitations of this study. Our study addresses a research gap in liver disease and participating communities. There is a lack of prior research on viral hepatitis/liver cancer needs in Michigan, as this work has not been previously conducted in the state. When similar studies were implemented elsewhere, the study populations were concentrated in mostly urban and large cities as opposed to smaller locations like Hamtramck and Battle Creek in West Michigan. However, our outreach methods may have limited the inclusivity of our study, as research has shown that those who are more engaged in the community tend to also be more proactive about their health or experience better self‐rated health [30, 31]. Furthermore, the results of this study were not intended to be generalisable to other Asian American communities, as factors such as state policies and community member age can affect determinants. Instead, we hope our methods can be used as a toolkit to understand determinants in other areas, which can then inform adapted chronic disease education and screening interventions.

After culminating a series of validated sources into a comprehensive survey, we administered it to Burmese, Bengali and Chinese communities across Michigan to quantitatively analyse their viral hepatitis screening determinants. Overall, individuals who had previously been tested for viral hepatitis tended to be wealthier, more proficient in English, and had immigrated to the U.S. earlier. They also demonstrated higher health literacy, greater social support and better knowledge of HBV transmission. However, similarities and differences in determinants emerged. Chinese community members reported the least number of barriers to HBV/HCV screening, as opposed to Burmese community members, who struggled with health literacy and Bengali community members, who were unlikely to engage in health‐seeking behaviours. Informed by the results and the CAPs feedback, we will develop a culturally tailored intervention to address specific HBV/HCV education and screening needs.

## Author Contributions

P.W.: writing – original draft preparation (with support from N.D.P. and P.V.P.). N.D.P. and P.V.P.: conceptualisation; support for writing – original draft preparation. J.T.: formal analysis. H.P., T.C., Q.Z. and Y.‐C.W.: investigation. T.‐Y.W.: resources.

## Funding

This work was supported by Rogel Cancer Center, University of Michigan.

## Conflicts of Interest

The authors declare no conflicts of interest.

## Data Availability

The authors confirm that the data supporting the findings of this study are available within the article.
